# Serotonin transporter genotype modulates functional connectivity between amygdala and PCC/PCu during mood recovery

**DOI:** 10.3389/fnhum.2013.00704

**Published:** 2013-10-31

**Authors:** Zhuo Fang, Senhua Zhu, Seth J. Gillihan, Marc Korczykowski, John A. Detre, Hengyi Rao

**Affiliations:** ^1^Department of Psychology, Sun Yat-Sen University, GuangzhouChina; ^2^Center for Functional Neuroimaging, Department of Neurology, University of PennsylvaniaPhiladelphia, PA, USA; ^3^Center for the Treatment and Study of Anxiety, Department of Psychiatry, University of PennsylvaniaPhiladelphia, PA, USA

**Keywords:** 5-HTTLPR, amygdala, PCC/PCu, functional connectivity

## Abstract

The short (S) allele of the serotonin transporter-linked polymorphic region (5-HTTLPR) has been associated with increased susceptibility to depression. Previous neuroimaging studies have consistently showed increased amygdala activity during the presentation of negative stimuli or regulation of negative emotion in the homozygous short allele carriers, suggesting the key role of amygdala response in mediating increased risk for depression. The brain default mode network (DMN) has also been shown to modulate amygdala activity. However, it remains unclear whether 5-HTTLPR genetic variation modulates functional connectivity (FC) between the amygdala and regions of DMN. In this study, we re-analyzed our previous imaging dataset and examined the effects of 5-HTTLPR genetic variation on amygdala connectivity. A total of 15 homozygous short (S/S) and 15 homozygous long individuals (L/L) were scanned in functional magnetic resonance imaging (fMRI) during four blocks: baseline, sad mood, mood recovery, and return to baseline. The S/S and L/L groups showed a similar pattern of FC and no differences were found between the two groups during baseline and sad mood scans. However, during mood recovery, the S/S group showed significantly reduced anti-correlation between amygdala and posterior cingulate cortex/precuneus (PCC/PCu) compared to the L/L group. Moreover, PCC/PCu-amygdala connectivity correlated with amygdala activity in the S/S group but not the L/L group. These results suggest that 5-HTTLPR genetic variation modulates amygdala connectivity which subsequently affects its activity during mood regulation, providing an additional mechanism by which the S allele confers depression risk.

## Introduction

Recent psychiatric research has focused increasingly on understanding both genetic and neural mechanisms associated with differential vulnerability to mood and anxiety disorders (for reviews see Hariri and Holmes, 2006; Canli and Lesch, 2007). The serotonin transporter-linked promoter region (5-HTTLPR), a specific polymorphism of the serotonin transporter gene (SLC6A4), has been one of the most widely studied genetic variations in the last decade and appears to be an important modulator of emotional behavior. The 5-HTTLPR is in the promoter region upstream from the serotonin transporter gene and comprises both short (S) and long (L) variants, with significantly reduced transcriptional efficiency and serotonin uptake in the S allele compared to the L allele (Lesch et al., 1996).

Behavioral research has well demonstrated a significant association between 5-HTTLPR variability and differential vulnerability to mood and anxiety disorders. For e.g., Lesch et al. (1996) first reported that S allele carriers displayed higher levels of anxiety-related traits than homozygous long individuals (L/L), which was replicated by several subsequent studies (Mazzanti et al., 1998; Greenberg et al., 2000). Furthermore, Caspi et al. (2003) demonstrated that individuals carrying the S allele exhibited more depressive symptoms, diagnosable depression, and suicidality in response to stressful life events than L allele homozygotes, which was also replicated by other studies (Kaufman et al., 2004; Kendler et al., 2005; but see Risch et al., 2009; Klumpers et al., 2012). In addition, previous studies have also identified significant effects of 5-HTTLPR genetic variation on human physiology including cortisol responses (Gotlib and Hamilton, 2008; Chen et al., 2009; Reimold et al., 2011).

By integrating brain imaging and genetics, genomic neuroimaging studies have begun to define the neurobiological pathways whereby the 5-HTTLPR increases risk for depression, particularly through its effects on amygdala activity (Hariri et al., 2002; Canli et al., 2005). Using neuroimaging techniques including both positron emission tomography (PET) and functional magnetic resonance imaging (fMRI), previous studies have consistently demonstrated amygdala dysfunction in depression and other psychiatric disorders. For e.g., previous PET studies have shown abnormally increased amygdala metabolism in depression (Drevets et al., 1992, 2002; Abercrombie et al., 1998). Dys-regulation of amygdala function has been implicated in several affective and mood disorders (Phillips et al., 2003). Recent fMRI studies have also shown amygdala hyper-activity both at rest and in response to negative stimuli among individuals with depressive disorders (Brockmann et al., 2011; Zhong et al., 2011). Moreover, amygdala hyperactivity in depressed patients may be normalized by antidepressant treatment, which often target serotonin neurotransmitters (Godlewska et al., 2012; Rosenblau et al., 2012). For the relationships between 5-HTTLPR genotype and amygdala activity, Hariri et al. (2002) first reported that S allele carriers showed greater amygdala reactivity in response to fearful or angry faces than did L allele homozygotes. A number of independent studies have replicated the incremental effects of the S allele on amygdala activation during negatively valenced or stressful conditions compared to neutral conditions (Bertolino et al., 2005; Canli et al., 2005; Heinz et al., 2005; Brown and Hariri, 2006; Dannlowski et al., 2008; Gillihan et al., 2011). Several perfusion imaging studies also reported greater resting amygdala activity associated with the S allele (Canli et al., 2006; Rao et al., 2007; but see Viviani et al., 2010). The intriguing similarity of amygdala hyper-activity among depressed individuals and healthy S allele carriers at rest and in response to negative events suggests that differential patterns of amygdala reactivity may be a neural pathway mediating individual differences in vulnerability to depression.

Genetic variation of 5-HTTLPR not only modulates regional amygdala activity, but also alters functional coupling between amygdala and other emotion regulation regions, particularly amygdala-prefrontal cortex (PFC) connectivity. Many studies of healthy adults have shown that medial PFC (mPFC) moderates amygdala activity, which plays an important role in coping with subjective distress (Quirk et al., 2003; Likhtik et al., 2005; Zhou et al., 2012). Pezawas et al. (2005) reported decreased functional coupling between the amygdala and subgenual cingulate cortex in S allele carriers, which inversely predicted temperamental anxiety. In contrast, Heinz et al. (2005) found greater positive coupling between the amygdala and ventromedial PFC in S allele carriers, which may contribute to amygdala hyper-activity. Similarly, Friedel et al. (2009) showed increased functional connectivity (FC) between the amygdala and mPFC associated with the S allele in healthy individuals; however, the correlation was reversed in patients with major depressive disorder. Hariri and Holmes (2006) have proposed an integrated model and suggested that 5-HTTLPR genotype variation modulates the neural emotion regulation network by altering PFC-amygdala functional coupling that may subsequently change amygdala reactivity.

As a key structure for emotion processing and regulation in the brain, the amygdala is functionally connected not only to the anterior cingulate and prefrontal cortices, but also to other brain regions including the parahippocampal gyrus, insula, and posterior cingulate cortex/precuneus (PCC/PCu; Stein et al., 2007; Roy et al., 2009; Robinson et al., 2010; Zhu et al., 2012). The PCC/PCu is a core region of the brain default mode network (DMN), a network that is more active at rest than during goal-oriented tasks (Raichle et al., 2001). Greater DMN activity while viewing negative pictures has been found in depressed patients compared to control subjects (Sheline et al., 2009, 2010), suggesting that depressed individuals may use different strategies to process negative emotional stimuli. As the DMN is involved in self-referential activities (Seminowicz et al., 2009; Sheline et al., 2009), greater DMN activity in depressed patients may reflect that depressed individuals experienced more inappropriate self-referential thoughts while viewing the negative pictures. A number of studies have demonstrated negative functional coupling between the amygdala and PCC/PCu activity (Stein et al., 2007; Roy et al., 2009; Veer et al., 2011). However, it remains unknown whether 5-HTTLPR genetic variation modulates FC between the amygdala and PCC/PCu that may subsequently alter amygdala activity. Therefore, in the present study, we re-analyzed our previous imaging dataset (Gillihan et al., 2010) to examine the effects of 5-HTTLPR genetic variation on amygdala connectivity during resting baselines, sad mood, and mood recovery, respectively. Our study used the emotional experience of loss (imagine loss of a loved one) to induce participants’ depressive feelings which has been successfully used to predict depression onset (Kendler et al., 2003). We hypothesized that the homozygous short individuals (S/S) group would show a different amygdala connectivity pattern compared to L/L group during mood regulation. Specifically, given the previous findings of increased amygdala activity during mood recovery for the homozygous S/S group compared to the L/L group (Gillihan et al., 2010); we anticipated that the S/S group would show significantly reduced anti-correlations between the amygdala and PCC/PCu during mood recovery, which subsequently alters amygdala activity. We also anticipated that the S/S group would show significantly increased FC between the amygdala and mPFC.

## Methods

### Participants

**Figure 1:**
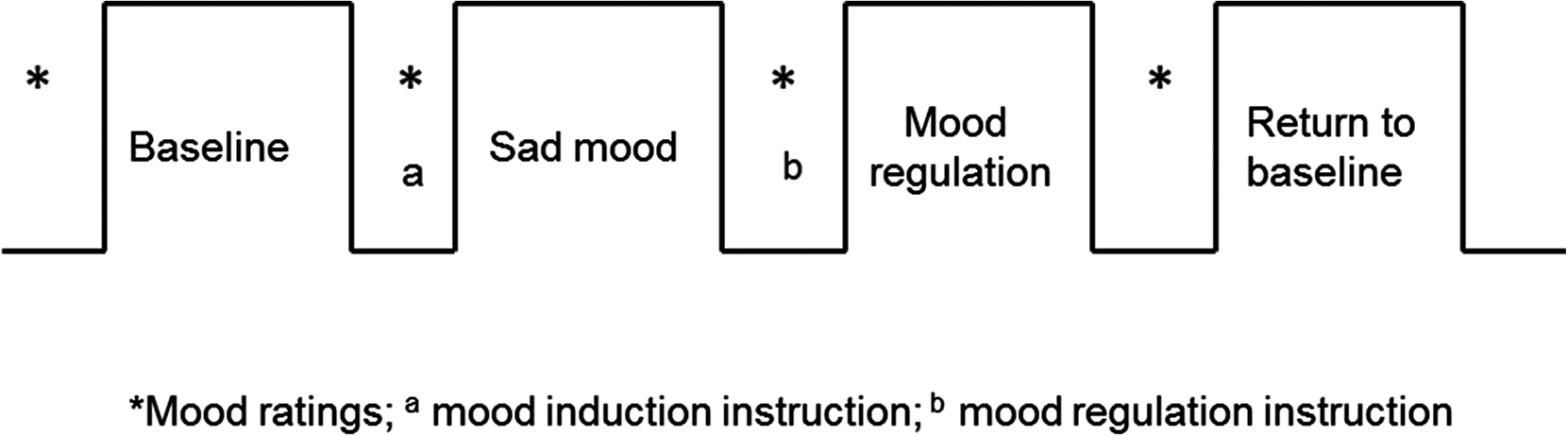
**The fMRI study design.** The sad mood scan began once participants indicated that they had reached their saddest mood; the mood regulation scan began immediately following the mood regulation instructions.

All participants were recruited by responses to study flyers. A total of 275 participants were screened for 5-HTTLPR genotype and self-reported ethnic background. Within this large cohort, 15 homozygous short (S/S) and 15 homozygous long (L/L) healthy Caucasian individuals participated in this brain imaging study (16 males; mean age 20.3 years, range 18–29 years). For the purpose of isolating the effects of each allele, we limited our investigation to homozygotes and did not include the heterozygous genotypes in the study. Non-Caucasian participants were excluded due to potential differences in allelic frequencies as a function of ancestry (Malhotra and Goldman, 1999). Participants also completed behavioral measures of depression symptoms and personality (See below). All the participants were given a psychiatric interview Structured Clinical Interview for DSM-IV (SCID) to screen out those with a current psychiatric diagnosis and none of the participants had neurological illness or current psychiatric diagnosis. The fMRI scans are the final testing session, which included four functional runs (Figure 1): first task-free resting baseline scan (no task), induced sad mood scan, mood regulation scan, and a second task-free scan following return to baseline. Participants were asked to rate their moods (sadness and anxiety) before and after each functional scan. Anxiety ratings were obtained to make sure that any observed neural differences between the genotype groups were not induced by the possible differences in anxiety between the genotype groups, as there is evidence that the S allele is associated with anxiety-related traits (Lesch et al., 1996). All the participants provided written informed consent in accordance with the Institutional Review Board of the University of Pennsylvania. They received monetary compensation for participation in the study.

### 5-HTTLPR genotyping

Each participant provided two buccal cell samples for genotyping, scraping one Whatman^®^ Sterile Omni swab (Fisher Scientific) against the inside of each cheek for 30 s. Swabs were air dried for 2 h. Genomic deoxyribonucleic acid (DNA) was prepared from buccal cells using the Qiagen QIAamp^®^ Blood Mini Kit (Qiagen, Inc, Valencia, California). Forward (5'-ATG CCA GCA CCT AAC CCC TAA TGT-3') and reverse (5'-GG ACC GCA AGG TGG GCG GGA-3') primers were used to amplify a fragment from the serotonin transporter promoter region. These primers amplify a 419 base pair fragment for the 16-repeat L allele and a 375 base pair fragment for the 14-repeat S allele (Gelernter et al., 1997). Polymerase chain reaction (PCR) was carried out on a Reaction Module (BioRad iCycler, #170–872), and the products were separated on a 2.5% agarose gel (Agarose SFR, Amresco Inc., Solon, Ohio) supplemented with Ethidium Bromide (0.01%, Fisher Scientific) and visualized under ultraviolet light.

### Behavioral measures

The behavioral measures included the Beck Depression Inventory (BDI; Beck et al., 1996) to assess depression symptoms; the NEO-Five-Factor Inventory (NEO-FFI; Costa and McCrae, 1992) to assess the major personality dimensions; and the SCID (First et al., 1996) to assess current and lifetime psychiatric diagnoses.

### fMRI scans, mood tasks and ratings

All participants completed four fMRI scans: a resting baseline scan, a sad mood scan after induction of the sad mood, a mood regulation scan while trying to get out of the sad mood, and another resting baseline scan after the return to baseline mood. The duration of each scan was 6 min. Participants were asked to rate their moods from 0–100 (lower indicates worse mood) for both anxiety and sadness before and after each of the four scans (See Figure 1).

#### Baseline scans

During resting baseline scans, participants received the following instruction: “Lie still and let your mind go blank, but keep your eyes open and stay awake.”

#### Sad mood induction

During the sad mood induction procedure, participants were asked to imagine vividly the death of a living, healthy loved one. The experimenter read the following brief script that asked participants to imagine specific aspects of losing their loved one and to really experience any sad feelings as they imagined the death scenario: “Now I’m going to ask you to think vividly about something that would make you really sad; specifically, think about losing a loved one. Picture the person in their dying moments. You might imagine trying to comfort them but not really succeeding; you might imagine their terror or despair. Picture this scene as vividly and concretely as possible. Think about where the person is. Think about the clothes they’re wearing. Picture their hair, their face, and their eyes. Imagine their voice and what they might be saying. They’re scared and they don’t want to die. You might also imagine the final goodbye, as your loved one is laid to rest: the coffin closing, the family and friends who are there but can’t ease your pain; the burial, the body of your loved one lowered into the ground, where they will remain forever. The person you loved cannot come back, no matter how dearly you wish it; the person is there, in the ground, forever. Really allow yourself to experience any negative feelings that these images bring up for you.” Participants squeezed a rubber bulb that triggered a tone to alert the experimenter when they had reached their saddest mood; the experimenter then obtained anxiety and sadness ratings. The sad mood scan began immediately thereafter.

#### Mood regulation instruction

For mood regulation, participants received the following instruction: “Now use whatever technique you can think of to get yourself out of this sad mood. You might try to question the soundness of your negative thoughts that you would not be able ultimately to adjust and cope with this loss. You could imagine yourself getting all the support you need from family and friends to deal with it. You might imagine your loved one going about their day, healthy, happy, and safe. Think about the support you have in your life. If despite all this your sad mood is still persisting, feel free to turn your mind to more positive scenes or memories to either distract yourself from or to neutralize the sadness as much as possible.”

### Data acquisition and analysis

All participants were scanned on a Siemens 3.0 Tesla Trio whole-body scanner (Siemens AG, Erlangen, Germany), using a standard Transmit/Receive head coil. Concurrent perfusion and BOLD data were acquired using the continuous arterial spin labeling (ASL) technique with a gradient echo-planar imaging (EPI) sequence with the following parameters: FOV = 22 cm, matrix = 64 × 64, TR = 3 s, TE = 17 ms, label time = 1.6 s, delay time = 0.8 s, flip angle = 90°. Fourteen slices (8 mm thickness with 2 mm gap) were acquired from inferior to superior in sequential order. Before the functional scan, high-resolution anatomical images were obtained by a 3D Magnetization Prepared Rapid Acquisition Gradient Echo (MPRAGE) sequence with TR = 1620 ms, TI = 950 ms, TE = 3 ms, flip angle = 15°, 160 contiguous slices, 1 × 1 × 1 mm^3^ resolution.

In the present study, we focused on the FC analysis of the EPI data from the ASL scans. Imaging data processing and analyses were carried out with the Statistical Parametric Mapping software (SPM8, Wellcome Department of Cognitive Neurology, UK) and REST 2.0 toolbox^1^, implemented in Matlab 14 (Math Works, Natick, MA). For each participant, functional images were realigned to correct for head motion, coregistered with the anatomical image, and normalized to a 3 × 3 × 3 mm^3^ Montreal Neurological Institute (MNI) template using bilinear interpolation. The normalized functional images were smoothed using a Gaussian filter with a full-width at half maximum (FWHM) of 4 mm and the band-pass ranges between 0.01–0.08 Hz. Six head motion parameters, the global mean signal, white matter signal, cerebrospinal fluid (CSF) signal as well as an additional covariate for the ASL labeling ([1,−1 … 1, −1]) were entered as covariates into the regression model and submitted to the FC analyses.

Bilateral amygdala was used as the seed region for FC analyses. The amygdala region of interest (ROI) was determined a *priori* from an automated anatomical labeling (AAL) ROI library (Tzourio-Mazoyer et al., 2002) in the SPM Marsbar toolbox (Brett et al., 2002). The FC maps of the four scan blocks were created for each participant in two genotype groups and transformed into Fisher’s *Z* in order to improve the normality before entering the group-level analysis. One-sample *t*-tests were performed to examine the overall pattern of amygdala connectivity across all scan blocks for both genotype groups as well as for each scan block of each genotype group. Paired *t*-tests were also performed to examine the differences between the two genotype groups for each scan block. For the overall pattern of amygdala connectivity, activation clusters were identified at a significance level of whole brain family-wise error (FWE) corrected *p* < 0.05. For comparisons between the two genotype groups, activation clusters were identified at a significance level of voxel-wise uncorrected *p* < 0.001 and cluster size larger than 10 voxels (270 mm^3^), which corresponded to a whole brain Alphasim corrected *p* < 0.05. Because we have specific hypotheses on the PCC/PCu and mPFC, we also applied the small volume correction based on the independently defined PCC/PCu and mPFC ROIs from the literature. The PCC/PCu ROI was defined as a sphere with 10 mm radius located in the MNI coordinate [0, −50, 31] (Greicius et al., 2003, Zhu et al., 2013), while the mPFC ROI was defined as a sphere with 10 mm radius located in the MNI coordinate [0, 52, −3] (Heinz et al., 2005; Friedel et al., 2009).

Because ROI analyses comprise many fewer statistical comparisons relative to voxel-wise analyses, and therefore present less risk of a Type I error, we also conducted ROI analyses based on independently defined ROIs to test our hypotheses. The strength of FC between the amygdala and PCC/PCu were extracted for statistical comparisons. In addition, in order to test the hypothesis that increased amygdala activation in the S/S group during mood regulation may be associated with reduced negative correlation between amygdala and PCC/PCu, we carried out correlation analyses between amygdala-PCC/PCu connectivity and regional cerebral blood flow (CBF) in the amygdala ROI, which was defined by the peak amygdala activation cluster from the contrast between mood regulation and resting baselines (MNI coordinate [22, −8, −20]) in the S/S group (Gillihan et al., 2010).

## Results

### Behavioral

As reported in our previous study (Gillihan et al., 2010), the two groups were matched and there were no differences in age, gender, depression symptoms, or personality dimensions between the two genotype groups (all *p* > 0.4, Table 1). As shown in Table 2, both groups showed significant decreases in sadness rating scores (indicating worse mood) after sad mood induction compared to baseline (both *p* < 0.001). Furthermore, at the end of the sad mood scan, the mood rating scores were still low in both groups, suggesting that saddest mood lasted through the whole sad mood scan. After the mood regulation scan, the mood rating scores were significantly increased (indicating better mood) in both groups (both *p* < 0.001), supporting the effectiveness of mood recovery. However, there was no effect of 5-HTTLPR genotype variation on the changes of mood ratings (*p* = 0.15–0.95), suggesting that any significant difference in neural activity is unlikely to be due to subjective differences in mood.

**Table 1:** **Demographic, depression symptom and personality scores by genotype**.

**Variable**	**L group mean (SD)**	**S group mean (SD)**	***p*-value**
Age	20.6(2.6)	20.0(1.4)	0.44
Female/Male	6.9	8.7	0.46
BDI	7.1(6.4)	6.5(5.6)	0.81
Neuroticism	31.3(6.5)	30.8(6.5)	0.82
Extraversion	41.9(6.0)	40.5(7.1)	0.56

*The two genetic groups were similar on age, gender, depression symptoms, and personality dimensions. BDI, Beck Depression Inventory*.

**Table 2:** **Mood ratings before and after fMRI scans by genotypes**.

	**Mood rating scores**				
	**Baseline**	**Sad-Start**	**Sad-End**	**Post-Reg**	**End**
L group mean (SD)	73.0(16.8)	30.0(13.4)	36.1(19.5)	66.3(14.6)	71.3(13.2)
S group mean (SD)	79.7(12.9)	35.0(15.3)	36.5(16.8)	73.3(13.8)	77.9(11.0)
*p*-value	0.23	0.35	0.95	0.19	0.15

*Sad-Start: the start of the sad mood scan after the subject reporting the saddest mood. Sad-End: the end of the sad mood scan. Post-Reg: the score after the mood regulation. The higher rating score means the better mood*.

### Neuroimaging

**Figure 2:**
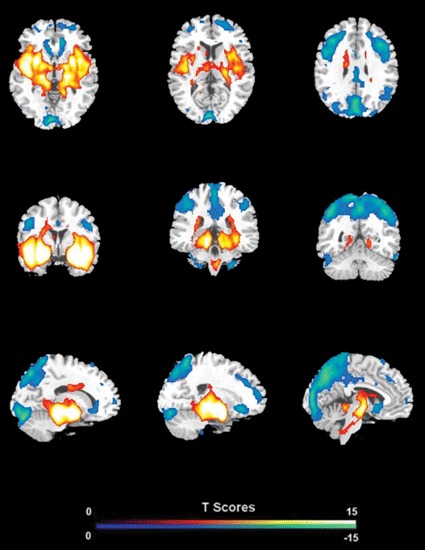
**The overall pattern of amygdala connectivity from whole brain analysis.** Threshold was set as whole brain FWE corrected *p* < 0.05.

**Figure 3:**
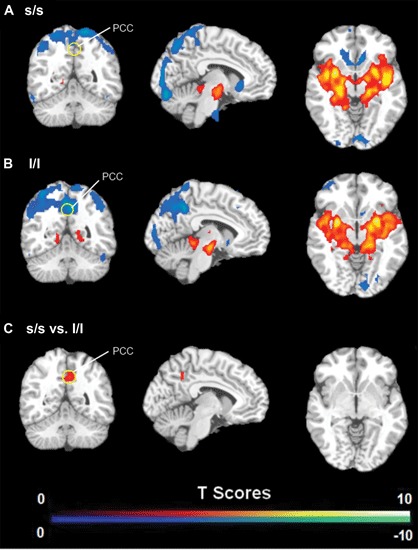
**(A)** Amygdala connectivity for the s/s group during the mood recovery scan. **(B)** Amygdala connectivity for the l/l group during the mood recovery scan. **(C)** Amygdala connectivity differences between the s/s and l/l groups during the mood recovery scan. Threshold was set as uncorrected *p* < 0.001. The PCC/PCu differences survived small volume corrected *p* < 0.05.

FC analysis revealed an overall pattern of amygdala connectivity similar to previous studies (Stein et al., 2007; Roy et al., 2009; Robinson et al., 2010; Veer et al., 2011). Specifically, the amygdala showed positive connectivity to the basal ganglia, insula, para-hippocampal/hippocampal gyrus, temporal cortex, and subgenual cingulate cortex, and negative correlation to the PCC/PCu, occipital cortex, parietal cortex, and dorsal and medial frontal regions (Figure 2). Comparisons between the S/S and L/L genotype groups revealed no between-group differences in amygdala connectivity during the first baseline, sad mood, and return to baseline scans. However, during the mood recovery scan block, the S/S group showed significantly reduced negative correlation between amygdala and PCC/PCu (peak MNI coordinates *X* = 3, *Y* = −51, *Z* = 39, small volume corrected *p* = 0.021; Alphasim corrected *p* < 0.05; Figure 3). However, no connectivity differences were found between amygdala and mPFC in the two genotype groups.

**Figure 4:**
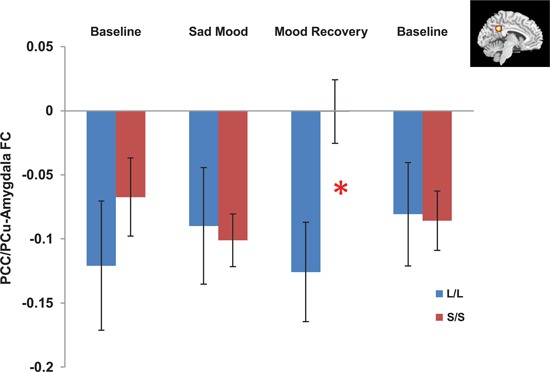
**Independent ROI analyses showed significant differences in the negative functional connectivity (FC) between the posterior cingulate cortex/precuneus (PCC/PCu, red region in the right corner) and amygdala between the s/s and l/l groups for the mood recovery scan, but no differences for the baseline and sad mood scans.** *p < 0.05.

The independent ROI analysis on the PCC/PCu confirmed the findings from whole brain analysis. No differences in PCC/PCu-amygdala connectivity were found between the two genotype groups for the baseline and sad mood scans, while significant differences in PCC/PCu-amygdala connectivity were found for the mood recovery scan (*p* = 0.017, Figure 4). However, the mPFC ROI analysis showed no differences between the two genotype groups for all scans.

**Figure 5:**
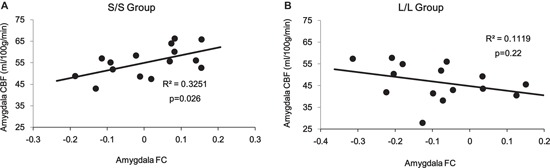
ROI analyses showed positive correlation between PCC/PCu-amygdala connectivity and amygdala regional CBF in the s/s group **(A)** but no significant correlation in the l/l group **(B)** during the mood recovery scan.

Correlation analyses on the amygdala ROI revealed a significant interaction (*p* = 0.014) between 5-HTTLPR genotype and PCC/PCu-amygdala connectivity on regional amygdala CBF that was selectively increased in the S/S group during mood recovery. The S/S group showed a significant positive correlation between PCC/PCu-amygdala connectivity and amygdala CBF (*r* = 0.57, *p* = 0.026, Figure 5A), while the L/L group showed a non-significant negative correlation between PCC/PCu-amygdala connectivity and amygdala CBF (*r* = −0.34, *p* = 0.22, Figure 5B).

## Discussion

By applying FC analysis to our previously acquired data, the current study demonstrated a similar pattern of connectivity between amygdala and multiple cortical and limbic regions in both *s/s* and *l/l* groups in four sessions, including positive connectivity to the basal ganglia, insula, para-hippocampal/hippocampal gyrus, temporal cortex and subgenual cingulate cortex, and negative correlation to the PCC/PCu, occipital cortex, parietal cortex, and dorsal medial frontal regions. These results are consistent with findings from previous studies (Pezawas et al., 2005; Stein et al., 2007) and suggest that the connections between these regions and amygdala are involved in implementing an important emotional network.

To our knowledge, the present study is the first to demonstrate the significant effect of 5-HTTLPR genetic variation on FC between amygdala and PCC/PCu, which is linked to mood regulation. We found significantly reduced negative functional coupling between amygdala and PCC/PCu in the S/S group compared to the L/L group during recovery from a sad mood, whereas no differences were found during sad mood and the baseline scans. In addition, the differential amygdala connectivity during mood regulation did not change when we reanalyzed the data without global signal regression, indicating that these findings in amygdala-PCC/PCu anti-correlations are not artifacts from global signal regression (Murphy et al., 2009; Saad et al., 2012).

A number of studies have consistently suggested the important role of PCC/PCu activity in emotion-related processes, including emotion evaluation (Wright et al., 2008), happy and sad words processing (Maddock et al., 2003), and social behavior (Adolphs, 2003). Several studies showed that individuals with greater PCC/PCu activation might be able to resolve a depressive mood more easily (Mayberg et al., 1999, 2005). Our findings are consistent with this literature and suggest that PCC/PCu was negatively coupled to amygdala during mood regulation in the L/L but not S/S individuals. Using structural equation modeling, a study on a large human fMRI data set has identified negative effective connectivity between PCC and amygdala (Stein et al., 2007). Negative functional coupling between amygdala and PCC/PCu activity has also been observed in healthy people and individuals with mood disorders (McClure et al., 2007). These findings provide converging evidence supporting the important role of PCC/PCu in modulating amygdala activity during mood regulation. In the current study, we found a weaker negative functional coupling between the amygdala and PCC/PCu in the S/S group during mood recovery, suggesting a deficient modulation from PCC/PCu on amygdala activity in the S/S group compared with L/L group. The differential amygdala-PCC/PCu anti-correlations may also reflect differential mood regulation strategies in the S/S versus L/L individuals.

By integrating amygdala connectivity with regional CBF data, we further observed a significant positive correlation between amygdala-PCC/PCu connectivity and amygdala CBF in the S/S group but not in the L/L group during the mood recovery scan. This positive correlation suggests that decreased amygdala-PCC/PCu connectivity is linked to higher amygdala CBF for the S/S carriers. These results are consistent with our previous finding that amygdala activity for the S/S group was significantly greater than the L/L group during the mood regulation scan (Gillihan et al., 2010). One explanation for this finding is that PCC/PCu may serve as one of the inhibition nodes of amygdala activity. For S/S carriers, down-regulation from the PCC/PCu to the amygdala was significantly reduced, leading to amygdala hyper-activity. This finding is also consistent with studies of social anxiety disorder showing that the PCC modulates amygdala activity (Pessoa et al., 2005; Hahn et al., 2011). The reduced negative correlation between the amygdala and PCC/PCu in the S/S group during sad mood regulation provides another neural mechanism underlying the increased vulnerability to mood and anxiety disorders for the S/S individuals, particularly in response to stressful life events.

Altered amygdala-PCC/PCu FC in the S/S group also supports the potential involvement of DMN in mood regulation. Large regions in the DMN are involved in autobiographical memory and rumination, and the presence of rumination may be a risk factor for the onset and maintenance of depression (Zhu et al., 2012). Altered DMN function has been found in depressed individuals compared to healthy controls during the performance of emotion tasks (Gotlib and Hamilton, 2008; Sheline et al., 2009). Decreased FC in the PCC/PCu was also observed in clinically depressed individuals (Zhu et al., 2012). The current study extends these findings by demonstrating the modulation effect of 5-HTTLPR genotype variation on the negative correlation between DMN and amygdala.

In contrast to the significant effect of 5-HTTLPR genetic variation on amygdala-PCC/PCu connectivity, we did not observe a significant effect of 5-HTTLPR genetic variation on amygdala-mPFC connectivity that has been reported in previous studies (Heinz et al., 2005; Pezawas et al., 2005). This apparent inconsistency may be due to differences in the subject population and emotional tasks used in the studies. In our study, we screened a large sample of 275 participants and selectively scanned 15 homozygous short and 15 homozygous long subjects matched for age, gender, depression symptoms, and personality scores. However, in previous studies, both homozygous and heterozygous genotypes were included in the study. Furthermore, previous studies employed negative emotional stimuli such as aversive pictures or fear faces. Although briefly viewing such negatively valenced stimuli can reliably activate the amygdala, at least in the L/L individuals, they do not bear a substantial resemblance to the events that have been reported to interact with 5-HTTLPR genotype to predict depression onset, like the experience of loss (Kendler et al., 1998). In the current study, we used a relatively more ecological task to induce a sad mood by the imagined loss of a loved one, which replicated the loss experience and induced relative real sadness as evidenced by the self-ratings and by the tears that several participants shed. In addition, there are some methodological differences between the present and previous studies that may also contribute to the inconsistent results. The present study used the concurrent BOLD signal from ASL perfusion imaging, while the previous studies used the conventional BOLD signal. Although a very recent study (Zhu et al., 2013) has demonstrated that both concurrent and conventional BOLD signals can be used in FC analysis, these two types of BOLD signals are different in signal-to-noise ratios and spatial and temporal resolutions, which may account for different connectivity findings.

The present study also has several other limitations. First, genotype-related differences in amygdala connectivity during sad mood were not observed. This may be due to the experimental design in which we did not scan the participants during the sad mood induction. The S/S and L/L groups may be different in the initial responses to sad and stressful events. Future work is needed to examine the neural mechanisms underlying the transition from a neutral mood to sadness. Second, the sample size of the current study is relatively small and we excluded individuals with heterozygous genotypes. Further work should include both homozygotes and heterozygotes with a larger sample size. Third, we asked the participants to imagine the death of a loved one to induce sad mood, which undoubtedly diminished the magnitude of the perceived loss compared to the actual death of a loved one. Fourth, the sad mood changes were assessed by the self-reported mood rating scores. Future studies should include more objective measurements like physiological or performance changes to examine the effects of sad mood induction and mood regulation. Finally, the spatial resolution of the concurrent BOLD signal from ASL perfusion imaging in the present study is relatively poor thus cannot distinguish the heterogeneous amygdala structure. Future studies with higher spatial resolution are needed to examine the effects of 5-HTTLPR genotype on the distinct FC patterns of subregions within the amygdala (Mishra et al., 2013).

In summary, the present study replicates previous findings that activity in the amygdala is anti-correlated with activity in the PCC/PCu, a key region of DMN that facilitates mood regulation. Further, this study shows that 5-HTTLPR genotype modulates activation and communication in this neural circuit during recovery from sad mood. The negative correlation between amygdala and PCC/PCu was significantly reduced in individuals with the S/S genotype compared to individuals with the L/L genotype, which was associated with greater amygdala activity during mood regulation in the S/S group. These findings may represent another potential mechanism whereby the S allele confers higher depression risk.

## Conflict of interest statement

The authors declare that the research was conducted in the absence of any commercial or financial relationships that could be construed as a potential conflict of interest.

## Footnotes

^1^http://resting-fmri.sourceforge.net/
